# Supplementary material for: Enrichment of Water Bodies with Phenolic Compounds Released from Betula and Pinus Pollen in Surface Water
Source: Plants (Basel). 2023 Dec 28;13(1):99. doi: 10.3390/plants13010099 (PMC10780553; doi:10.3390/plants13010099)
Supplement: Supplementary file 1 [file plants-13-00099-s001.zip › plants-2740978-supplementary.pdf]

SUPPLEMENTARY MATERIAL

Enrichment of Water Bodies with Phenolic Compounds Released from Betula and Pinus Pollen in Surface Water

Kerienė I., Šaulienė I., Šukienė L., Judžentienė A., Ligor MG. Valiuškevičius, Grendaitė D. and Buszewski B.

Table S1. Meteorological conditions of the last 10 days at the meteorological stations closest to the sampling location.

Meteorological station	Sampling date	Last 10 days meteorological conditions			
		Air temperature average, °C	Total precipitation, mm	Wind speed average, m/s	Prevailing wind directions
Klaipėda	2022-04-29	7.9	0.0	2.8	W-NW
	2022-06-06	11.7	29.2	2.9	SE
Šiauliai	2022-05-04	7.9	8.2	2.2	NW
	2022-05-06	8.4	10.1	2.5	NW
	2022-05-10	9.2	1.9	1.7	NW
	2022-06-04	13.2	66.1	2.2	SE
	2022-06-08	15.0	74.8	2.7	SE

The lakes and artificial inland water bodies described in the article are located in the north-western part of Lithuania near Šiauliai—the meteorological conditions of their catchments are represented by the data of the Šiauliai meteorological station. The meteorological conditions of the Curonian Lagoon and the Baltic Sea coast in the western part of Lithuania are described using the data of the closest Klaipėda meteorological station.

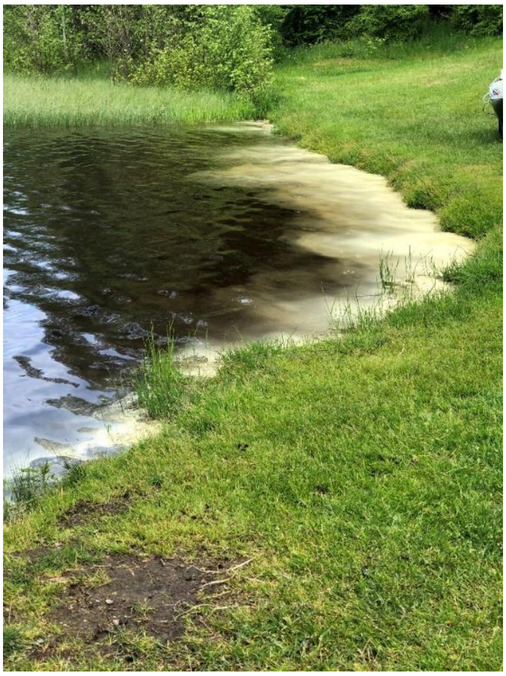

Figure S1. Aglomerates of the *Pinus* pollen in the surface water of natural lake Geluva (NLG).

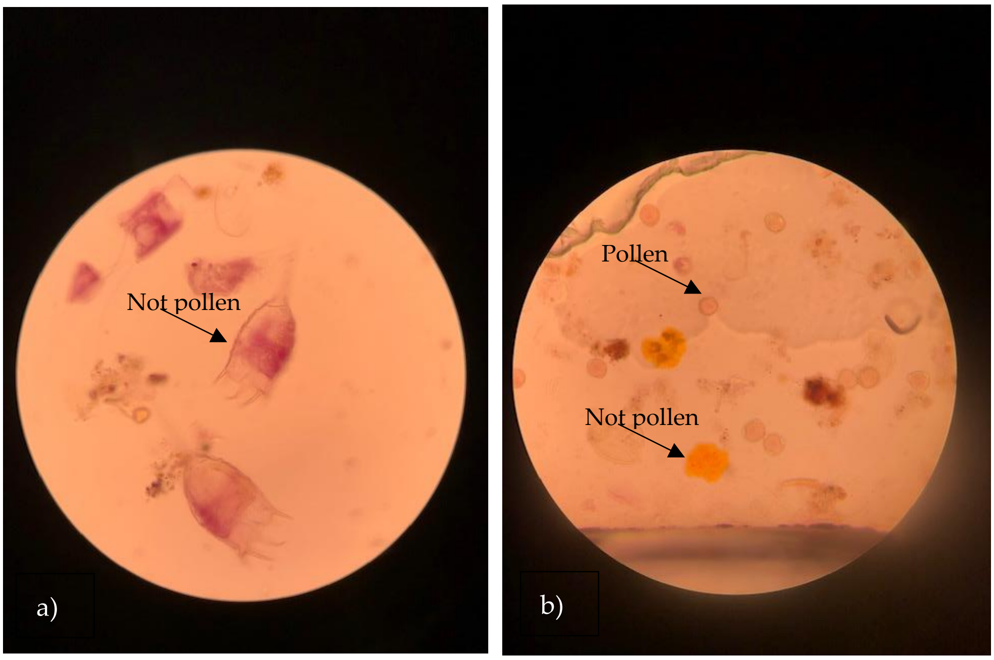

Figure S2. Water-suspended particles view through microscopy during the *Betula* pollen spreading period in flooded quarry Kalniškiai (FQK) (**a**) and dammed natural lake Juodlė (DNL) (**b**).

Table S2. Bioactivity of water-suspended particles and surface water during *Betula* and *Pinus* pollen spreading period.

Trophicity Status	Water Body	Antioxidant Activity	
		9.0–13.0% (ABTS•)	16.0–18.0% (DPPH•)
Dystrophic	NLS	SPB SPP SPPW	SPBW
Mesotrophic	FQK	SPB	
Mesotrophic-eutrophic	DNLB	SPP	
Eutrophic-hypereutrophic	DNLJ	SPB	SPBW
Hypereutrophic	CLC	SPB SPBW	
Hypereutrophic	DFD	SPB SPBW	

Meaning of abbreviations: NLS–natural lake Šermuksnynas; FQK–flooded quarry Kalniškiai; DNLB –dammed natural lake Bijotė; DNLJ–dammed natural lake Juodlė; CLC–surface water of the Curonian Lagoon coast; DFD –dammed fishpond Damba. SPB–water-suspended particles collected during Betula pollen spreading period; SPP–water-suspended particles collected during Pinus pollen spreading period; SPBW–surface water samples collected during the Betula pollen spreading period; SPPW–surface water samples collected during the Pinus pollen spreading period.

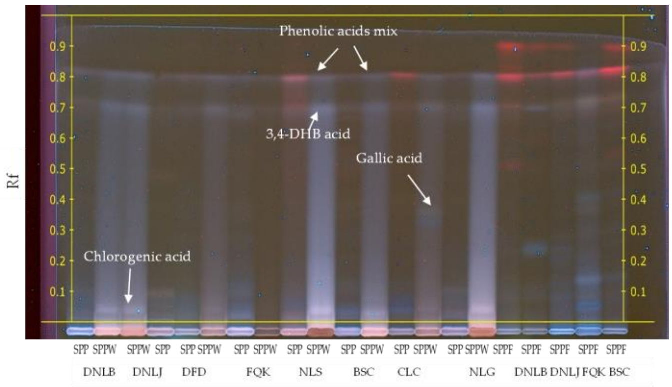

Figure S3. HPTLC of water-suspended particles and surface water during *Pinus* pollen spreading period. Track line: SPP—water-suspended particles collected during *Pinus* polen spreading period

(bound PC); SPPW—surface water samples collected during Pinus pollen spreading period; SPPF—water-suspended particles collected during *Pinus* pollen spreading period (free PC). Injection volume 15.0 µl. Mobile phase: chloroform/ethyl acetate/acetone/formic acid 40/30/20/10 *v/v/v/v*.

Table S3. Statistical results according to Kruskal-Wallis, Friedman rank sum tests and Dunn's test with Bonferroni correction for *p*-values of the total phenolic content in *Betula* and *Pinus* pollen exposed to distilled water for the appropriate time.

TPC	DSPB		DSPP		DSPBW **		DSPPW **	
	Kruskal-Wallis rank sum test							
	K-W	*p*	K-W	*p*	K-W	*p*	K-W	*p*
Control, 24 h, 48 h, 72 h	14.11 *	0.003	13.00 *	0.005	1.58	0.45	8.00*	0.02
	Friedman rank sum test							
	Friedman χ^2	*p*	Friedman χ^2	*p*	Friedman χ^2	*p*	Friedman χ^2	*p*
Control, 24 h, 48 h, 72 h	9.30 *	0.03	5.10	0.16	1.34	0.72	1.00	0.80
	Dunn's Test with Bonferroni correction for p-values							
	Z	p.adj	Z	p.adj	Z	p.adj	Z	p.adj
Control and 24 h	1.19	1.00	1.19	1.00	-	-	-	-
Control and 48 h	2.38	0.10	3.23 *	0.007	-	-	-	-
Control and 72 h	3.60 *	0.002	2.71 *	0.04	-	-	-	-
24 h and 48 h	1.19	1.00	2.04	0.25	−1.23	0.66	−1.96	0.15
48 h and 72 h	1.19	1.00	−0.52	1.00	0.84	0.40	2.75 *	0.02
24 h and 72 h	2.38	0.10	1.52	0.77	−0.39	1.00	0.78	1.00

Meaning of abbreviations: TPC—total phenolic content, DSPB—phenolic compounds in *Betula* pollen exposed to distilled water for the appropriate time, DSPBW—phenolic compounds in distilled water in which the *Betula* pollen was exposed for the appropriate time. DSPP—phenolic compounds in *Pinus* pollen exposed to distilled water for the appropriate time, DSPPW—phenolic compounds in distilled water in which the *Pinus* pollen was exposed for the appropriate time. **—control not applied. * Significant difference at $p < 0.05$. P.adj—adjusted p-values after Bonferroni correction.

Table S4. Statistical results according to Kruskal-Wallis, Friedman rank sum tests and Dunn's test with Bonferroni correction for *p*-values of the antioxidant activity of Betula pollen exposed in distilled water for the appropriate time.

TPC	DPPH_ DSPB		DPPH_ DSPBW		ABTS _ DSPBW		ABTS _ DSPB	
	Kruskal-Wallis rank sum test							
	K-W	*p*	K-W	*p*	K-W	*p*	K-W	*p*
24 h, 48 h, 72 h	6.50 *	0.04	4.31	0.12	6.62 *	0.04	9.85 *	0.007
	Friedman rank sum test							
	Friedman χ^2	*p*	Friedman χ^2	*p*	Friedman χ^2	*p*	Friedman χ^2	*p*
24 h, 48 h, 72 h	8.20 *	0.04	1	0.80	3.40	0.33	8.20 *	0.04
	Dunn's Test with Bonferroni correction for p-values							
	Z	p.adj	Z	p.adj	Z	p.adj	Z	p.adj
24 h and 48 h	2.15	0.09	1.96	0.15	0.98	0.98	1.57	0.35
48 h and 72 h	−2.26	0.07	−1.57	0.35	−2.55 *	0.03 *	1.57	0.35
24 h and 72 h	−0.10	1.00	0.39	1.00	−1.57	0.35	3.14	0.005

Meaning of abbreviations: TPC—total phenolic content , DSPB—phenolic compounds in *Betula* pollen exposed to distilled water for the appropriate time, DSPW—phenolic compounds in distilled water where the *Betula* pollen was exposed for the appropriate time. * Significant difference at $p < 0.05$. P.adj—adjusted p-values after Bonferroni correction.

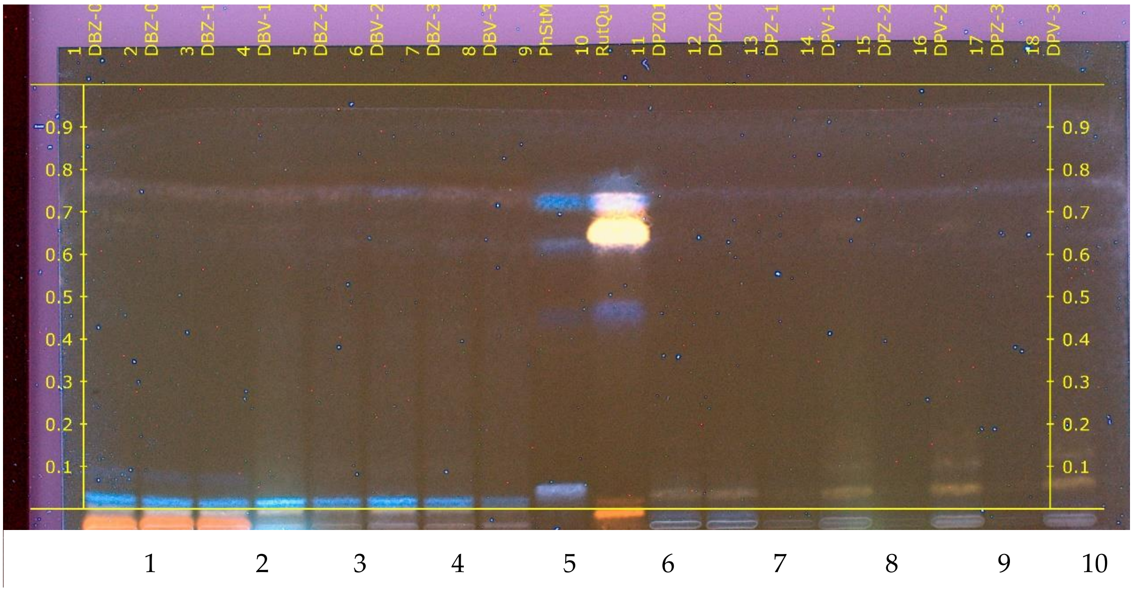

Figure S4. High-performance thin layer chromatography of *Betula* and *Pinus* water-suspended pollen in distilled water extracts after derivatization of DPPH• solution. Track indications: 1–2 control (Betula pollen collected in situ). Betula pollen exposed to distilled water: 3—for 24 h (DSPB1); 5—for 48 h (DSPB2); 7- for 72 h (DSPB3). Distilled water where *Betula* pollen was exposed: 4—for 24 h (DSPBW1); 6- for 48 h (DSPBW2); 8—for 72 h (DSPBW3). 9—phenolic acids standard mix; 10—rutin and quercetin standards mix. 11-12—control (*Pinus* pollen collected in situ). *Pinus* pollen exposed to distilled water: 13—for 24 h (DSPP1); 15—for 48 h (DSPP2); 17—for 72 h (DSPP3). Distilled water where Pinus pollen was exposed: 14—for 24 h (DSPPW1); 16—for 48 h (DSPPW2); 18—for 72 h (DSPPW3).Mobile phase consisted of chloroform/ethyl acetate/ acetone/formic acid (40/30/20/10 *v*/*v*/*v*/*v*).Injection volume: samples 10 µL, standards 2 µL.

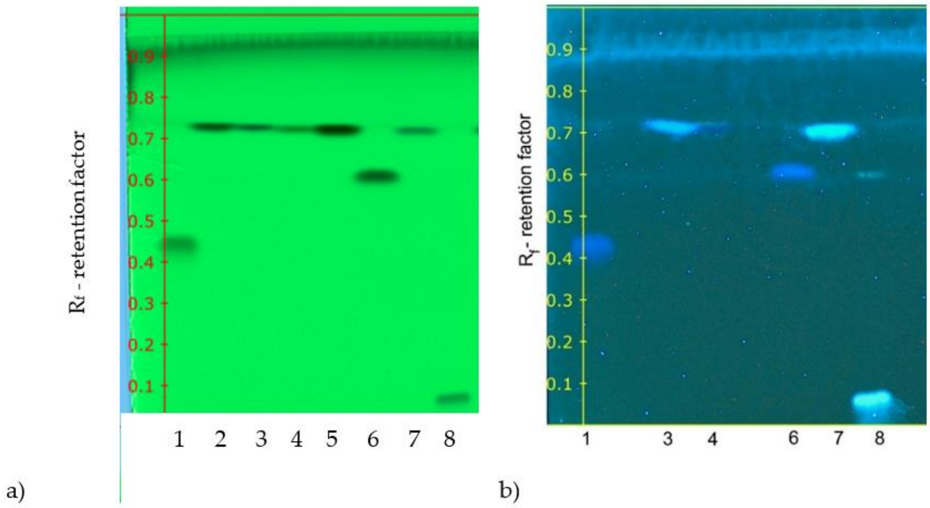

Figure S5. HPTLC of phenolic acid standards before (**a**) and after derivatization of DPPH• solution (**b**). Glowing lines show: 1—gallic acid; 2- vanillic acid, 3—*trans*-ferulic acid; 4—*p*-coumaric acid; 5—p-hydroxybenzoic acid, 6—3,4-dihydroxybenzoic acid; 8—chlorogenic acid. Mobile phase consisted of chloroform/ethyl acetate/acetone/formic acid (40/30/20/10 *v*/*v*). Injection volume 2.0 µl.

Table S5. The calibration data of individual phenolic compounds for analysis by HPLC-DAD.

Reference standard	UV absorbance λ_{max}, nm	Retention time, min	Regression equation	Determination coefficient, R^2	Limit of quantity, µg	Limit of detection (LOD), µg
Trans-ferulic acid	260	1.9	Y = 16106 x + 702	0.992	0.10–1.0	0.03
Vanillic acid	260	2.9	Y= 17417 x – 1382	0.992	0.25–1.0	0.10
Gallic acid	260	3.3	Y = 6548 x	0.999	0.25–1.0	0.10
p-Hydroxybenzoic acid	260	6.8	Y = 23139 x – 533	0.995	0.10–1.0	0.05
3,4-Dihydroxybenzoic acid	260	8.6	Y = 10179 x + 700	0.993	0.10–1.0	0.05
p-Coumaric acid	260	9.4	Y = 42392 x – 1827	0.997	0.10–1.0	0.05
Syringic acid	310	10.7	Y = 12455 x + 1873	0.899	0.10–1.0	0.03
Chlorogenic acid	310	11.1	Y = 39444 x + 647	0.999	0.10–1.0	0.03
Rutin	260	11.9	Y = 9874 x + 268	0.998	0.10–1.0	0.05
Quercetin	260	13.3	Y = 19236 x - 308	0.999	0.10–1.0	0.05
